# Finger Prosthesis Driven by DEA Pairs as Agonist–Antagonist Artificial Muscles

**DOI:** 10.3390/biomimetics9020110

**Published:** 2024-02-13

**Authors:** Alexandre B. S. da Silva, Gabriel E. P. Mendes, Eduardo S. Bragato, Guilherme L. Novelli, Marina Monjardim, Rafhael M. Andrade

**Affiliations:** 1 Department of Mechanical Engineering, Universidade Federal do Espírito Santo, Vitoria 29075-910, Brazil; 2 Graduate Program of Mechanical Engineering, Universidade Federal do Espírito Santo, Vitoria 29075-910, Brazil; 3 Graduate Program of Animal Biology, Universidade Federal do Espírito Santo, Vitoria 29075-910, Brazil

**Keywords:** finger, prosthetics, dielectric elastomer, artificial muscle

## Abstract

Loss of an upper limb exerts a negative influence on an individual’s ability to perform their activities of daily living (ADLs), reducing quality of life and self-esteem. A prosthesis capable of performing basic ADLs functions has the capability of restoring independence and autonomy to amputees. However, current technologies present in robotic prostheses are based on rigid actuators with several drawbacks, such as high weight and low compliance. Recent advances in robotics have allowed for the development of flexible actuators and artificial muscles to overcome the limitations of rigid actuators. Dielectric elastomer actuators (DEAs) consist of a thin elastomer membrane arranged between two compliant electrodes capable of changing dimensions when stimulated with an electrical potential difference. In this work, we present the design and testing of a finger prosthesis driven by two DEAs arranged as agonist–antagonist pairs as artificial muscles. The soft actuators are designed as fiber-constrained dielectric elastomers (FCDE), enabling displacement in just one direction as natural muscles. The finger prosthesis was designed and modeled to show bend movement using just one pair of DEAs and was made of PLA in an FDM 3D printer to be lightweight. The experimental results show great agreement with the proposed model and indicate that the proposed finger prosthesis is promising in overcoming the limitations of the current rigid based actuators.

## 1. Introduction

According to the Global Health Data Exchange of 2019 [1], for every 100,000 people worldwide, approximately 224 undergo the amputation of at least one upper limb, and 120 of them experience bilateral amputations of their limbs. The loss of an upper limb in the human body negatively impacts an individual’s ability to perform their activities of daily living (ADLs), reducing their quality of life and self-esteem. A prosthetic capable of carrying out basic daily functions becomes crucial in restoring independence and autonomy to amputees [2].

Current technologies in prosthetics and orthotics are based on rigid actuators [3], with limitations in the number of degrees of freedom, high weight, low flexibility, and low compliance with the prosthesis user [4,5]. Recent advances in robotics have allowed for the development of flexible actuators and artificial muscles with the intention of creating alternatives to overcome the limitations of rigid actuators [6].

**Figure 1:**
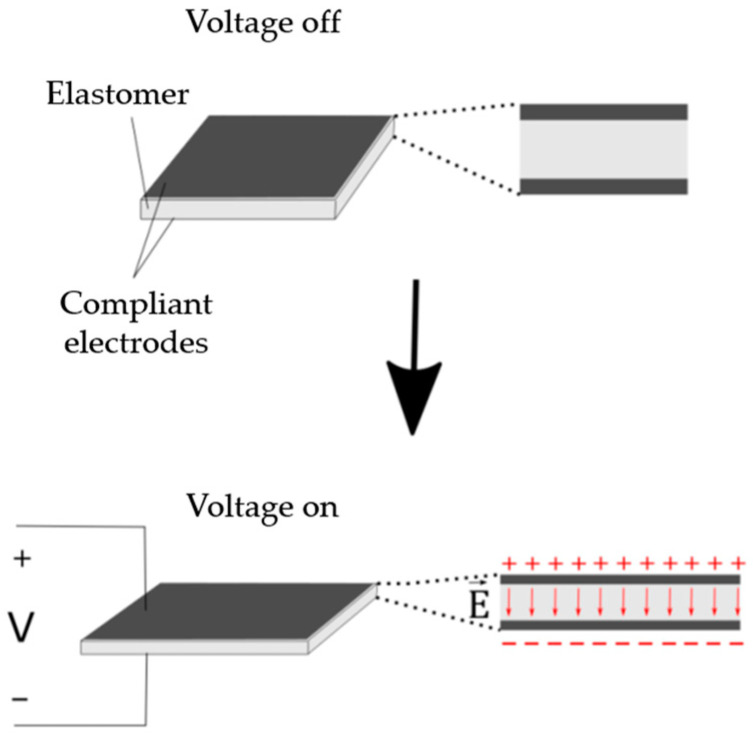
Rectangular dielectric elastomer.

Flexible actuators constitute a category of materials that respond to stimuli such as an electric field, temperature variation, concentration, and pH, thus altering their shape or dimensions [7]. Within the class of flexible actuators, there are electroactive polymers (EAPs), which are a type of polymer that changes its shape through electrical stimulation. Among EAPs, there are dielectric elastomers (DEs), which consist of a thin elastomer membrane positioned between two compliant electrodes (Figure 1), changing their dimensions when stimulated with an electric potential difference [8].

When a potential difference is applied between two electrodes, Maxwell stress arises in the elastomer, which begins to act as a dielectric. As the thickness of the elastomer can be reduced and the electrodes can approach each other, DEs act as electromechanical transducers, converting electrical energy into mechanical energy or vice versa. Among the DE applications, the most common are actuators, which convert electrical energy into mechanical energy [9], but there are also generators that transform mechanical energy into electrical energy [10], and sensors.

Compared to other flexible actuator technologies, DEs standout for their high deformation capabilities, high work densities (work done per actuation cycle normalized by the volume of the actuator), high specific power (output power value normalized by the mass of the actuator), and high efficiency. However, there are still practical difficulties that hinder the applications of DEs. The need for high electrical voltages for actuation; manufacturing difficulties; issues related to durability and maintenance; and the non-linearity of the transducer, which has a viscoelastic behavior, are the main constraints with this material [11].

Most of the muscles responsible for finger and wrist movements originate in the forearm and are called extrinsic muscles. These muscles are larger and provide force to movement, divided into the extensor and flexor muscles of the fingers, arranged in agonist–antagonist pairs. Although external, these muscles have insertions in the hand region to perform finger movements. Intrinsic muscles originate in the hand and are responsible for secondary movements, allowing fine and precise control of each finger [12,13].

Artificial muscles are defined as materials or devices that reversibly change shape and dimensions through external stimuli [7]. Among the external stimuli are the electric field for electroactive polymers and piezoelectric actuators, the temperature for shape memory alloys, the pressure for pneumatic actuators, and the magnetic field for magneto-rheological actuators [14], among other stimuli. Compared to other flexible actuators, dielectric elastomers stand out for their high deformation capacity and high work density [11].

In comparison with human striated skeletal muscles (Table 1), dielectric elastomer actuators (DEAs) exhibit superior characteristics in terms of maximum deformation, maximum tension, maximum deformation rate, work density, specific power, and efficiency [8,11,15,16,17,18].

The literature on artificial hands and fingers commonly addresses the design of artificial fingers that combine mechanics with embedded electronics, comprising tactile sensors for normal force and shear force [19,20]. Despite the use of new materials, sensors, and manufacturing processes in recent hand and finger prosthesis designs, such as the use of pneumatic artificial muscles [21], 3D-printed hand prosthetics [22], artificial muscles made from nylon threads [23], and prosthetics using stretchable optical waveguides [24], the use of flexible actuators to simulate agonist and antagonist muscles has not yet been explored and could benefit hand prosthesis design by simulating the movement of the natural limb [25].

This work presents the modeling, design, fabrication, and experimental validation of a finger prosthesis actuated by two fiber-constrained dielectric elastomer actuators (FCDEA) arranged in agonist–antagonist pairs to simulate natural finger movement. We designed an underactuated mechanism composed of two coupled four-bar chain mechanisms to allow for just one pair of FCDEAs to drive the finger. Since FCDEAs are linear expanding actuators [9,11], once an FCDEA expands, the agonistic pair contracts, thus rotating the driving rod of the mechanism. This approach mimics the skeletal striated muscles of the forearm to move the finger and introduces some advantages compared to other robotic hand prostheses, such as easier operation, noiselessness, mechanical compliance, and low weight. In Section 2, we introduce the materials and methods used to model, design, and manufacture the finger prototype and artificial muscles. Section 3 presents the results obtained with the physical prototype of the prosthetic finger. Finally, Section 4 discusses the conclusions derived from the project’s development and outlines the next steps in the project.

## 2. Materials and Methods

### 2.1. Finger FCDEA Setup

**Figure 2:**
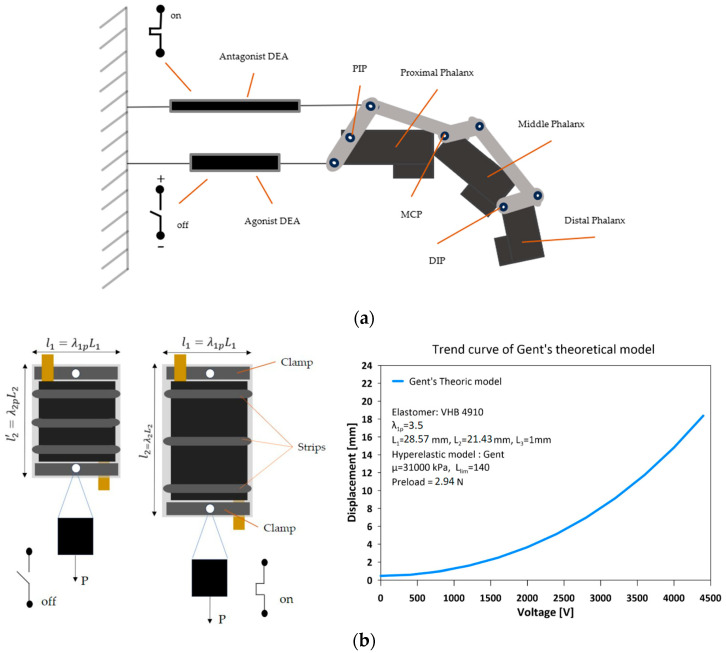
Finger prosthesis driven by two fiber-constrained dielectric elastomer actuators (FCDEA) displayed in antagonistic pairs. (**a**) Experimental setup for the FCDEAS composition and working principle of the finger prosthesis. (**b**) FCDEA activated and deactivated and displacement against voltage model.

The proposed finger prosthesis is driven by two FCDEA displayed in antagonistic pairs, as shown in Figure 2a. When activated, the DE membrane contracts in thickness and expands in area. The fibers constraint the expansion in one direction, thereby allowing the actuator increase length in just one direction, mimicking a skeletal striated muscle (Figure 2b).

In the human body, the movement of the limbs is provided by skeletal striated muscle arranged in antagonistic pairs. Once one muscle contracts, the antagonistic pair expands, thereby allowing for the joint moving. On the other hand, joint stiffness is controlled by the contraction intensity of the antagonistic pairs, i.e., greater joint stiffness is achieved by greater contraction of the muscles. The only difference from the proposed actuation system is that the FCDEAS expand upon actuation instead of contract. However, since it is arranged in antagonistic pairs, the functioning is the same. In other words, joint torque and displacement increases as the activation intensity difference of the antagonistic FCDEAS increases, and joint stiffness increases as the activation intensity of both antagonistic FCDEAS reduces.

The artificial muscle developed in this study was designed to replicate the contractile and extensible capacity of natural muscles, featuring a basic unit similar to a myofibril. Inspired by the efficient structure of sarcomeres, this type of artificial muscle aims to mimic the interaction between actin and myosin filaments to generate movement. In the FCDEA, the combination of two strips, along with the elastomer area, plays a functional role analogous to sarcomeres, while the strips mimic the Z-lines, allowing for an effective coordinated action of contraction and extension. Similarly to the muscular structure, increasing the number of FCDEA results in an increased tension that the artificial muscle can withstand. This innovative approach holds promising applications in fields such as robotics, biomechanical prosthetics, and assistive devices, where replicating muscular functionality is crucial for achieving precise and natural movements.

### 2.2. DEA Modeling

The strain energy density of the elastomer (WS) is obtained through material models known as hyperelastic models. For example, the Gent model considers the increase in stiffness at high deformations with a constant related to the stretch limit $J_{lim}$. Hoss and Marczak [26] cataloged more than twenty other hyperelastic models for various applications, while Wissler et al. [27] used some of these models to catalog the mechanical characteristics of an acrylic elastomer VHB 4010. Here, we highlight the Gent model [28], given by

(1) $$W_{s}=-\frac{\mu J_{lim}}{2}\ln\left(1-\frac{\lambda_{1}^{2}+\lambda_{2}^{2}+\lambda_{3}^{2}-3}{J_{lim}}\right)$$

where *m* is the shear modulus, $J_{lim}$ is the stretch limit constant, and *λ_i_* are the stretches in xyz directions.

To describe the state of the FCDEA, we should employ the hyperelastic Gent model and apply it to the original state equation of the standard elastomer. The constants used in these equations were determined for the VHB 4910, which was utilized in the experiments. Thus, the Gent model is redefined as follows:

(2) $$\sigma_{1}+\varepsilon {\left(\lambda_{1p}\lambda_{2}\frac{V}{L_{3}}\right)}^{2}=\frac{\mu \left(\lambda_{1p}^{2}-{\left(\lambda_{1p}\lambda_{2}\right)}^{-2}\right)}{1-(\lambda_{1p}^{2}+\lambda_{2}^{2}+{\left(\lambda_{1p}\lambda_{2}\right)}^{-2}-3)/J_{lim}}$$

(3) $$\lambda_{2}(\frac{P}{L_{1}L_{3}})+\varepsilon {\left(\lambda_{1p}\lambda_{2}\frac{V}{L_{3}}\right)}^{2}=\frac{\mu (\lambda_{2}^{2}-{\left(\lambda_{1p}\lambda_{2}\right)}^{-2})}{1-(\lambda_{1p}^{2}+\lambda_{2}^{2}+{\left(\lambda_{1p}\lambda_{2}\right)}^{-2}-3)/J_{lim}}$$

where *P* is the applied load; *σ*₁ represents the actual mechanical stress in the $L_{1}$ direction; $L_{1}$ and $L_{3}$ are the initial dimensions of the VHB 4910; ε is the electrical permittivity; *V* is the electrical voltage obtained from an external source; and $l_{1}=\lambda_{1p}L_{1}$, where $\lambda_{1p}$ represents the pre-stretching in the $L_{1}$ direction.

The Gent model demonstrates an increase in stiffness as the stretch increases. Therefore, a higher load or electrical voltage is required to achieve the same variation $\delta \lambda_{2}$ as the stretch increases. To estimate the displacement of the DEA as a function of the applied electrical voltage using the hyperelastic Gent model (Figure 2b), the properties of the VHB 4910 elastomer were used based on previous works [29,30,31]. A relative electrical permittivity of $\varepsilon_{r}=4.65$, a stretch limit constant of $J_{lim}=140$, and a shear modulus μ=31 kPa were considered. The applied stretch load P=2.94 N was adopted.

### 2.3. Finger Mechanism Modeling

**Figure 3:**
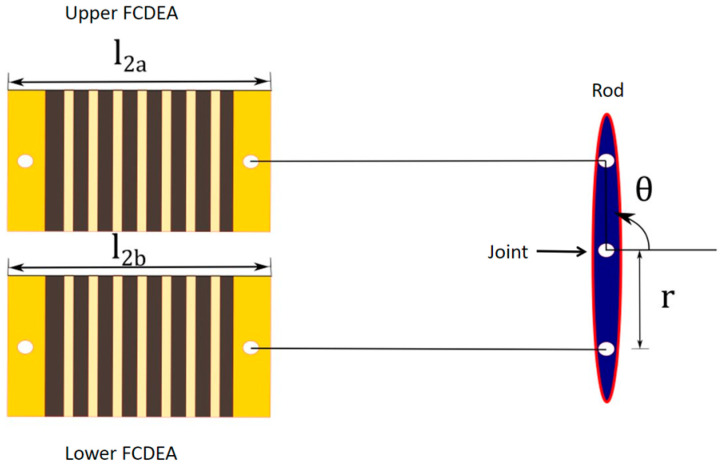
Underactuated mechanism to drive the finger prosthesis. The FCDAs are arranged in an antagonistic pair providing rod rotation.

The designed mechanism is an underactuated finger prothesis composed of two coupled four-bar chain mechanisms driven by two FCDEAs arranged in an antagonistic pair, as shown in Figure 3, where the expansion of the upper FCDEA provides clockwise rotation in θ, and the expansion of the lower FCDEA allows for anti-clockwise rotation in θ. The relationship between activation of the two FCDEAs is given by

(4) $$\theta =\sin^{-1}\left(\frac{L_{2}}{2r}\left(\lambda_{2b}-\lambda_{2a}\right)\right)$$

where *q* is the rotation of the first rod; *L*_2_ is the nominal dimension of the FCDEA in the actuation direction; and *l*_2*a*_ and *l*_2*b*_ are the stretches of the upper and lower FCDEA, respectively, which can be calculated accordingly by Equations (2) and (3).

**Figure 4:**
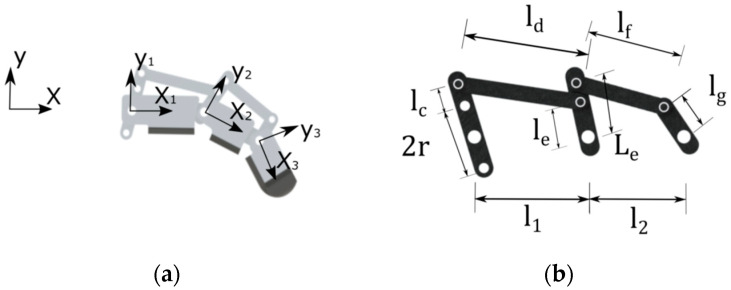
Prosthetic finger mechanism. (**a**) Bar mechanism; (**b**) bar mechanism.

The rotational movement of the first rod, as depicted in Figure 3, is responsible for driving the entire mechanism indicated in Figure 4. However, the complete finger movement has two components: the first one is responsible for moving the phalanges together (Figure 4a), and the second one is responsible for rotating the bar mechanisms around each phalange joint (Figure 4b).

**Figure 5:**
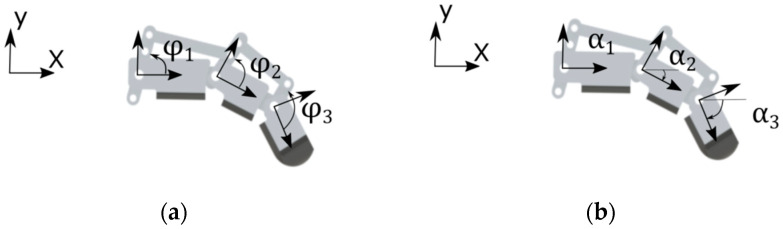
(**a**) Angles $\varphi_{i}$ and (**b**) angles $\alpha_{i}$.

To describe this movement, we must first introduce the angles $\varphi_{i}$ (Figure 5a), which indicate rod rotation relative to joint *i*, and the angles $\alpha_{i}$, (Figure 5b)**,** which show the rotation of the coordinate system ($X_{i},Y_{i}$) relative to the inertial coordinate system (X,Y).

In this way, there are two possible extreme movements that can be performed by the mechanism. The first one is the rotation of the first rod altering only the angles $\varphi_{i}$ (Figure 5a), and the second one is the rotation of the first rod, altering only the angles $\alpha_{i}$ (Figure 5b). To couple these two movements, a transmissibility coefficient $\tau_{i}$ is added to describe the amount of movement allocated to each motion. If this coefficient is defined as τ=1, the entire movement is carried out around the angles α, but if τ=0, the entire movement is carried out around the angles φ. Therefore,

(5) $$\delta \alpha_{i}=\tau_{i}\delta \theta_{i}$$

(6) $$\delta \varphi_{i}=\left(1-\tau_{i}\right)\delta \theta_{i}$$

To perform the movement of a finger with 1 degree of freedom capable of flexing all phalanges simultaneously, it is necessary that 0<τ<1. However, the transmissibility coefficient does not necessarily need to be constant throughout the movement. As noted, assuming a finger with a transmissibility coefficient equal to 1, all the phalanges flex with the same angle α. However, upon contact with an object on one of these phalanges, the rotation in α is restricted, thereby reducing the transmissibility coefficient to 0 once it is entirely directed to move the rods relative to each phalanx, altering φ.

Considering a voltage of 3500 V applied, the expected displacement of the DEA, according to the hyperelastic Gent model and Figure 2b, is about 11 mm. This displacement is used to estimate the dimensions and positions of the bars presented in Figure 4b, as shown in the Table 2 below.

### 2.4. FCDEA Finger Prototype

**Figure 6:**
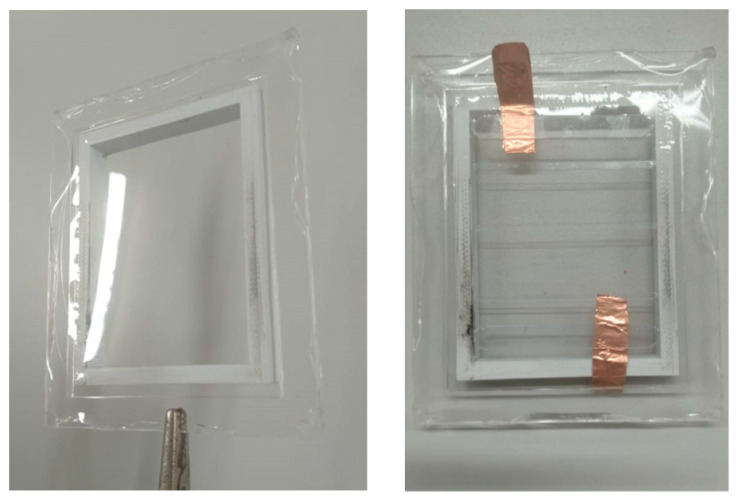

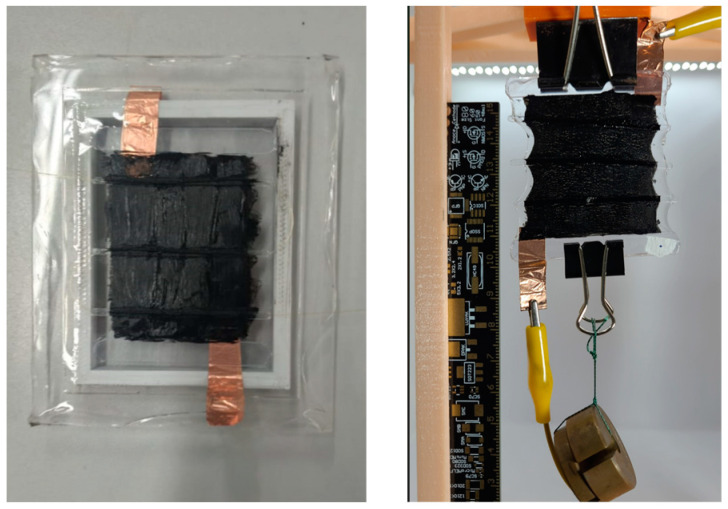
The step-by-step fabrication process of the DEA.

The step-by-step fabrication process of the FCDEAs is presented in Figure 6. Fabricating the actuator involves preparing the necessary materials, such as cutting PET fibers with a thickness of 0.20 mm; PET clamps with a thickness of 0.75 mm; and the VHB 4910 (3M, Maplewood, MN, USA) elastomer, which should be cut into a square with at least 64 mm on each side. Next, the elastomer is pre-stretched, where $\lambda_{p1}=\lambda_{p2}=3.5$. The acrylic frame is placed over the pre-stretched elastomer and pressed to improve adhesiveness. After that, the clamps, copper tapes, and PET fibers are attached onto the elastomer. Finally, carbon conductive grease (MG Chemicals, Burlington, ON, Canada) is applied to both surfaces of the VHB, and using a cutting tool, the DEA is detached from the acrylic frame. This results in a dielectric elastomer constrained by fibers. However, before its use, the elastomer needs to be suspended with a pre-load of 300 g for 30 min to enhance pre-stretching in the actuation direction. High voltage is provided by a DC-DC Converter 7000 V 3 W (E70, XP Power, Reading, Belgium).

The stretchable part of the elastomer membrane originally had dimensions of 64 mm × 64 mm. After stretching with $\lambda_{1p}=\lambda_{2p}=3.5$, we obtained new dimensions of 224 mm × 224 mm. The acrylic frame, with internal dimensions of 120 mm × 160 mm, is positioned tightly adhered to the VHB so that, upon detaching the acrylic frame from the pre-stretcher, the pre-stretching remains constant. The clamps and fibers support lateral pre-stretching $\lambda_{1p}=3.5$ throughout the actuation period of the FCDEA.

Two clamps, each 20 mm in height, and seven fibers, each 5 mm in height, are positioned, leaving approximately 10 mm of space (5 mm on each side) between the clamps and the acrylic frame. The dimensions of the free elastomer, without fibers or clamps, are 100 mm (160 mm − 2 × 5 mm − 2 × 20 mm − 7 × 5 mm = 100 mm) × 75 mm. These dimensions still have the same pre-stretching value $\lambda_{1p}=\lambda_{2p}=3.5$. Therefore, upon detaching the FCDEA from the frame, it tends to contract immediately in the actuation direction. Hence, the new nominal dimensions of the elastomers are as follows:

(7) $$L_{1}=\frac{l_{1}}{\lambda_{1p}}$$

(8) $$L_{2}=\frac{l_{2}}{\lambda_{1p}}$$

Thus, $L_{1}=28.57\;mm$ and $L_{2}=21.43\;mm$. For practical reasons, the length measurements of the elastomer during actuation were taken between the two clamps. Therefore, the sum of the heights of the fibers (7 × 5 mm = 35 mm) should be subtracted from *L*_2_ to find the actual values of $l_{2}$ and $\lambda_{2}$.

**Figure 7:**
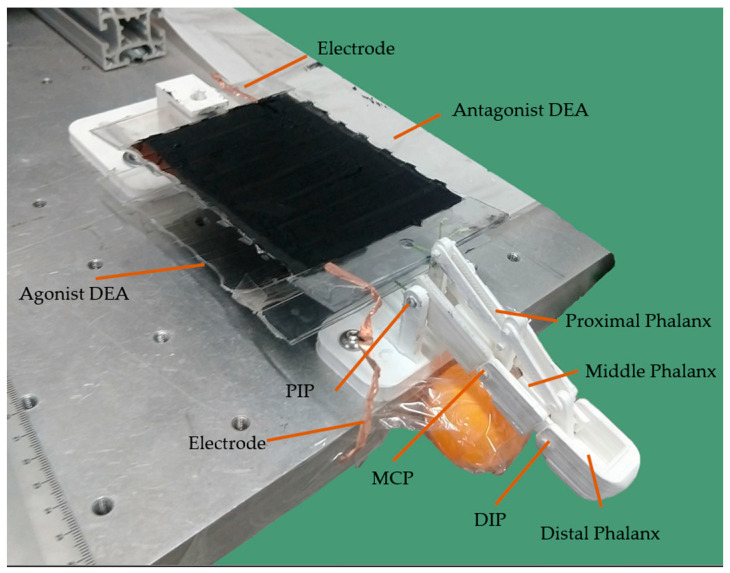
FCDEA finger prosthesis prototype assembled in a test bench.

The prosthetic finger was made of PLA by an FDM 3D printer (Sethi S3X, Sethi 3D, Campinas, SP, Brazil). Components were placed on an experimental bench to assess the bending and extension capacity of the prosthetic finger when actuated by the antagonistic pair of FCDEAs, as shown in Figure 7.

## 3. Results and Discussion

### 3.1. Finger Mechanism Simulation

**Figure 8:**
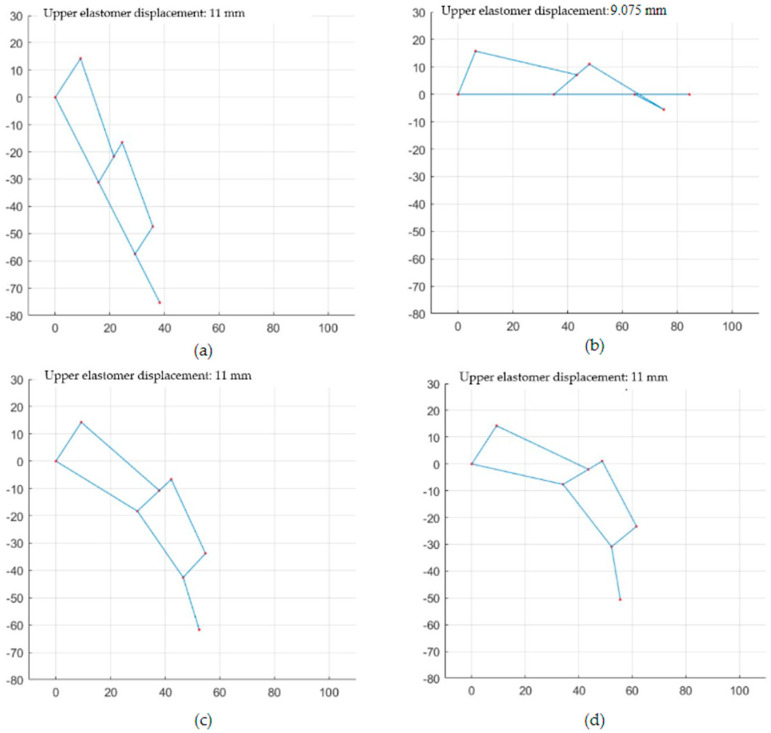
Prototype movement for (**a**) $\tau_{i}=1$ (**b**) $\tau_{i}=0$ (**c**) $\tau_{i}=0.5$ (**d**) $\tau_{1}=0.2$, $\tau_{2}=0.5$, $\tau_{3}=0.5$.

The movement of the underactuated prosthetic finger was estimated for different values of $\tau_{i}$, as shown in Figure 8, considering a displacement of the upper elastomer of 11 mm. Figure 8a presents the movement for $\tau_{i}=1$, while Figure 8b shows the movement for $\tau_{i}=0$. Since the second four-bar mechanism reaches its rotation limit when the elastomer displacement is greater than 9.075 mm, $\tau_{i}=0$ (Figure 8b), we did not provide accurate finger motion. For $\tau_{i}=0.5$, as shown in Figure 8c, the movement was equivalent to a real finger, performing interphalangeal rotation. Figure 8d presents the result for values of $\tau_{1}=0$, $\tau_{2}=0.5$, $\tau_{3}=0.5$.

### 3.2. Expandable Linear Actuator

**Figure 9:**
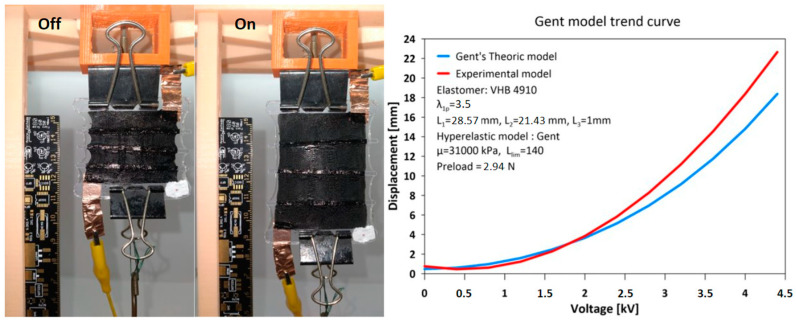
Electrical voltage curve at the source x average elongation of the actuators.

Five FCDEAs were manufactured for the purpose of testing the elongation as a function of the applied voltage. Figure 9 represents the average curve obtained from the manufactured actuators and the curve from the theoretical Gent model. After the 30 min period with preload, the actuators had an average initial length $l_{2_{0}}=96.4$ mm, and the maximum average displacement obtained was 27.4 mm for a supplied voltage of 4.8 kV, resulting in a maximum length of 123.8 mm.

Thus, the maximum and minimum stretches obtained in the actuation direction were

(9) $${\lambda_{2}}_{max}=\frac{123.8-35}{21.43}=4.14$$

(10) $${\lambda_{2}}_{min}=\frac{96.4-35}{21.43}=2.87$$

The maximum stretch provided by electrical actuation was

(11) $${\lambda_{actuator}}_{max}=\frac{123.8-96.4}{96.4}=0.287$$

Thus, the maximum deformation capacity of the FCDEA prototype was 28.4%, and the hyperelastic properties of the material was applied between elongations of 2.87 and 4.14. Since the manufactured FCDEAs had a greater displacement capacity than the Gent model, a voltage of 2700 V was considered to activate the FCDEA to obtain a displacement of 11 mm.

### 3.3. Physical Prototype

**Figure 10:**
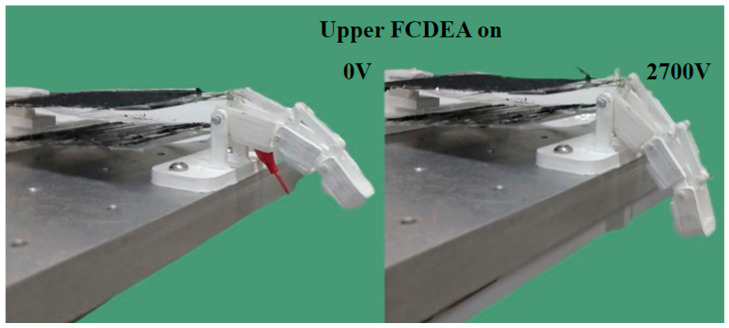
FCDEA finger prosthesis motion when the upper actuator was active.

The physical prototype of the prosthetic finger was tested on an experimental bench, presented in Figure 10, where the movement of the upper FCDEAs was activated with a voltage of 2700 V, resulting in a displacement of 11 mm.

Bending movement of the finger prototype was used to estimate values for $\tau_{i}$, by minimizing the angular error between the joint angle $\alpha_{i}$ and the experimental joint angle $\alpha_{i_{exp}}$:

(12) $$minimize\;\left(Error=\sum_{i=1}^{3}\alpha_{i}-\alpha_{i_{exp}}\right)$$

**Figure 11:**
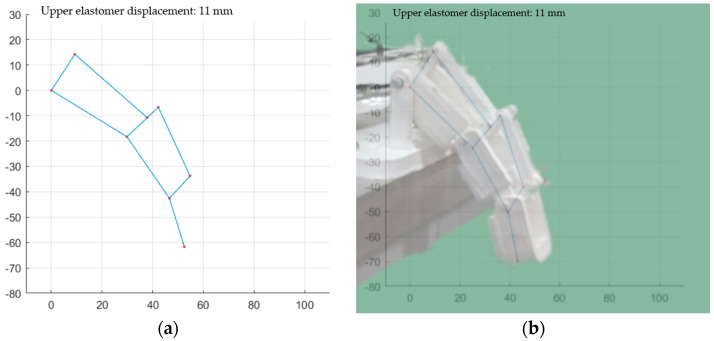
Model and prototype of the FCDEA finger prosthesis. (**a**) Model with transmissibility coefficients $\tau_{1}=0.7$, $\tau_{2}=0.6$, and $\tau_{3}=1$; (**b**) comparison between experiment and simulation movement.

The movement performed by the finger actuated by the antagonist pair closely resembles the model presented in Figure 11 when the values of the transmissibility coefficients $\tau_{1}=0.7$, $\tau_{2}=0.6$, and $\tau_{3}=1$. The simulation with these coefficients is depicted in Figure 11a, and the comparison of the actual movement with the simulated movement is shown in Figure 11b.

**Figure 12:**
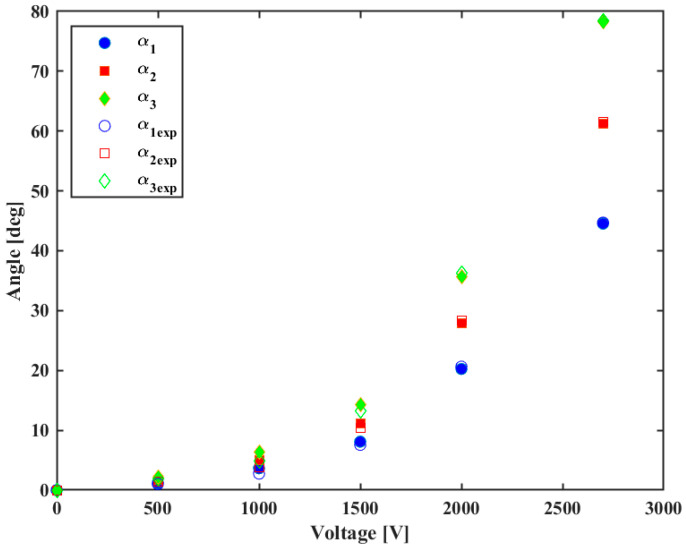
Estimated joint angles $\alpha_{i}$ and the experimental joint angles $\alpha_{i_{exp}}$.

Using the estimated transmissibility coefficients $\tau_{1}=0.7$, $\tau_{2}=0.6$, and $\tau_{3}=1$, it is possible to estimate the joint angles as a function of the input voltage, which may be used to control the position of the finger. Figure 12 presents the estimated the joint angle $\alpha_{i}$, filled symbols, and the experimental joint angle $\alpha_{i_{exp}}$, empty ones. The proposed model was quite accurate in estimating the joint angles of the finger prosthesis.

### 3.4. Gripping an Object

**Figure 13:**
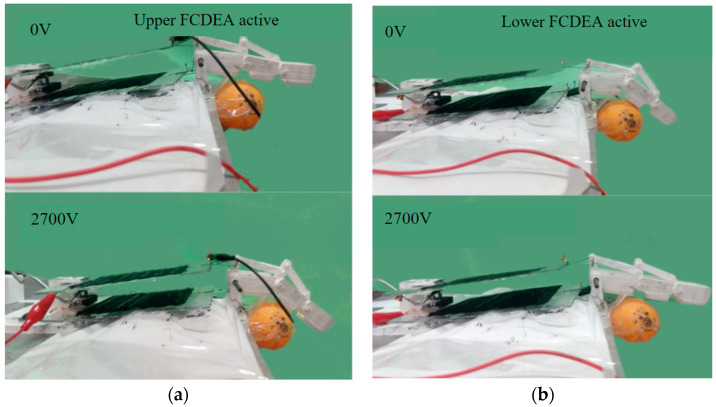
(**a**) Initial and final points of the flexion movement and (**b**) initial and final points of the extension movement.

Experiments were carried out to reproduce the flexion and extension motion of the finger around a small object, in this case, a table tennis ball. Figure 13a represents the complete flexion movement of the prototype around the object, including the initial and final positions, and Figure 13b illustrates the complete extension movement of the prototype. For flexion movements, the upper FCDEA was activated, while for extension movements, supply voltage was applied to the lower EDCF.

Figure 13 shows that the finger prothesis prototype driven by the FCDEA pairs as artificial agonist–antagonist muscles was able to mimic the behavior of a sound finger during flexion and extension movements around an object. Therefore, the same design concept can be used to build an FCDEA-driven full hand prosthesis to overcome some of the barriers faced by the current robotic hand prosthesis design, such as rigid actuators, noise, high weight, low flexibility, and low mechanical compliance [32,33]. Another possible design for the finger prosthesis could employ artificial tendons to move joints [13], where FCDEA can pull the tendons in an agonist–antagonist arrangement, as proposed here.

However, this study presents some limitations that are important to highlight. Our experimental approach considered only one FCDEA activated at a time to model the finger motion. But an agonist–antagonist pair of skeletal striated muscle achieves precise position, force, and stiffness control through co-contraction [34]. Moreover, the ability of human hands to perform precise operations is the result of multiple muscle synergies [35]. However, here, only one pair of FCDEAs was used to move the whole finger mechanism through the PIP joint. Therefore, to have a finger prosthesis model that is more faithful to the natural finger, it is necessary to regulate the activation of both agonist and antagonist FCDEAs at the same time and provide other pairs of artificial muscles to move the MCP and DIP joints independently.

Finally, dielectric elastomers possess promising properties in the field of rehabilitation robotics, such as being low weight, having a high energy density, and having a significant deformation capacity. However, there are still some barriers to their applications. The durability and maintenance capabilities of actuators that use dielectric elastomers remain extremely limiting factors for real-world applications. Additionally, the need for high voltages to actuate the elastomers is also a concern for human use. Recent advances have been overcoming these barriers and improving the design features of these actuators, suggesting that dielectric elastomers will likely find practical applications in robotics in the near future [7].

## 4. Conclusions

This work presented the modeling, design, fabrication, and experimental testing of a prosthetic finger actuated by two fiber-constrained dielectric elastomer actuators (FCDEA) arranged in antagonistic pairs as artificial muscles. The finger’s flexion movement is achieved when the upper FCDEA is activated, while the extension movement occurs when the lower actuator is engaged. The Gent hyperelastic model provided an appropriate estimated displacement, but some adjustments to the material parameters are still needed to precisely replicate the displacement of the manufactured FCDEA. A voltage of 2700 V was applied to achieve an 11 mm displacement of the elastomer and allow the full flexion and extension movement of the finger. The simulation results of the prosthetic finger showed results similar to those of the manufactured prototype. However, the actuator’s parameterization still needs optimization, conducting tests with different elastomer dimensions, applied loads, and pre-stretching, among others, to maximize deformation and load capacity and reach the ideal size for the proposed application. Additionally, dielectric elastomer actuators exhibit complex dynamic response properties that should be explored through oscillatory tests under different loading conditions to enable their use in conjunction with force and position controllers, something we intend to explore in future works.

## Figures and Tables

**Table 1:** Comparison between the performance of natural muscles and maximum values found in dielectric elastomers.

Metric	Skeletal Striated Muscle	Dielectric Elastomer
Maximum deformation (%)	40	142 (linear)
Maximum tension (MPa)	0.35	7.7
Maximum deformation rate (%/s)	50	450
Work density (kJ/m^3^)	40	3500
Specific power (kW/kg)	0.28	3.6
Efficiency (%)	40	80

**Table 2:** Dimensions of the FCDEA.

Metric	Elastomer
Eletrical Voltage (V)	3500
$\lambda_{1p}$	3.50
$L_{1}$ (mm)	28.57
$L_{2}$ (mm)	21.43
$l_{2}$ (mm)	11.00

## Data Availability

Data is unavailable due to privacy or ethical restrictions.
